# Effect of the Occlusal Splint Therapy on Mandibular Condylar Position in Patients With Myofascial Pain and an Average Skeletal Growth Pattern: A CBCT-Based Randomized Controlled Trial

**DOI:** 10.7759/cureus.119357

**Published:** 2026-11-09

**Authors:** Amol Karagir, Amit A Mhapuskar, Shridevi Adaki, Shams Ul Nisa, Darshan R Prasad Hiremutt

**Affiliations:** 1 Department of Oral Medicine and Radiology, Bharati Vidyapeeth (Deemed to be University) Dental College and Hospital, Pune, IND

**Keywords:** condylar position, cone-beam computed tomography, myofascial pain, occlusal splint therapy, pharmacotherapy, temporomandibular disorders

## Abstract

Introduction

Myogenic temporomandibular disorders (M-TMDs) cause masticatory muscle pain and impaired mandibular function. This study evaluated the effect of occlusal splint therapy on mandibular condylar position in patients with M-TMDs using cone-beam computed tomography (CBCT).

Materials and methods

This is a two-arm, open-label randomized controlled trial, with an additional non-randomized external healthy control group. A total of 65 participants were included; 44 patients with M-TMDs were randomly allocated to group A, which received occlusal splint therapy combined with pharmacotherapy (n=22), or group B, which received pharmacotherapy alone (n=22). Group C comprised 21 healthy participants recruited as a non-randomized reference group without TMDs. An average skeletal growth pattern was confirmed using composite cephalometric analysis. Condylar positional parameters, including the condylar ratio (CR), sagittal condylar guidance angle (SCGA), and temporomandibular joint spaces, were evaluated using CBCT. Groups A and B were assessed at baseline and after three months, whereas group C underwent a single baseline CBCT assessment. Data were analyzed using the Shapiro-Wilk test, paired sample t-test, one-way analysis of variance, and Tukey’s post hoc test.

Results

In group A, the right CR decreased significantly from 0.24 ± 0.10 at baseline to 0.11 ± 0.04 after three months (p<0.001). Significant within-group changes were also observed in the left CR (p=0.039), right SCGA (p=0.005), and left SCGA (p<0.001). Group A additionally demonstrated a greater reduction in pain intensity, increased maximum mouth opening, and significant changes in temporomandibular joint space measurements compared with group B. However, most post-treatment comparisons of CR and SCGA among the three groups were not statistically significant.

Conclusion

Three months of occlusal splint therapy combined with pharmacotherapy was associated with reduced pain, improved mouth opening, and short-term changes in CBCT-derived condylar positional parameters in patients with M-TMDs. These findings should be interpreted as positional adaptations rather than evidence of osseous condylar remodeling.

## Introduction

Myogenic temporomandibular disorders (M-TMDs), commonly characterized by myalgia or myofascial pain involving the masticatory muscles, constitute one of the most prevalent subtypes of temporomandibular disorders (TMDs), accounting for approximately 45.3% of diagnosed cases [1]. Their etiology is multifactorial and may involve biological, psychological, behavioral, and biomechanical factors. M-TMDs may also coexist with chronic pain conditions, including fibromyalgia and primary headache disorders. In patients with persistent symptoms, peripheral and central sensitization may contribute to pain that continues beyond the expected period of tissue healing [2]. Common clinical manifestations include pain or tenderness in the masticatory muscles, preauricular discomfort, restricted or deviated mandibular opening, and joint sounds such as clicking or crepitus. Muscle hyperactivity, altered neuromuscular control, clenching, and bruxism may additionally influence mandibular function and the spatial relationship of the mandibular condyle within the glenoid fossa [3].

Mandibular position is influenced by the functional relationship among the masticator muscles, dental occlusion, temporomandibular joints (TMJs), and craniofacial skeleton morphology. Changes in mandibular position may therefore affect the condylar orientation and joint-space relationship. Mandibular position is also anatomically related to the position of the tongue and surrounding upper-airway structures; however, associations identified using static imaging should not be interpreted as evidence of airway obstruction or altered respiratory function [4]. Craniofacial skeletal pattern may independently influence TMJ biomechanics, condylar position, and upper-airway dimensions. Restricting the study population to individuals with an average skeletal growth pattern may consequently reduce anatomical heterogeneity and improve the internal validity of comparison between treatment groups.

Cone-beam computed tomography (CBCT) enables a three-dimensional evaluation of osseous TMJ structures, condylar position, and joint-space measurements with high spatial resolution and a lower radiation dose than conventional computed tomography [5-8]. Nevertheless, CBCT has limited soft-tissue contrast and cannot directly assess the articular disc, masticatory-muscle activity, dynamic airway collapsibility, or respiratory function. Standardized patient positioning, image orientation, acquisition parameters, and anatomical landmarks are therefore necessary to obtain reproducible results and minimize methodological variability.

Occlusal stabilization splints are widely used as a reversible and conservative treatment for M-TMDs. By providing a stable occlusal surface and distributing occlusal forces more evenly, these appliances may reduce masticatory muscle activity, limit parafunctional loading, alleviate pain, and improve mandibular function [9-14]. Previous studies using CBCT have reported changes in condylar position and TMJ space relationship following stabilization splint therapy; however, their findings have been inconsistent [15-18]. Differences in study design, participant characteristics, skeletal morphology, splint materials and thickness, wearing schedule, treatment duration, imaging protocol, and landmark section may explain some of this variability. Evidence from randomized controlled trials evaluating CBCT-derived condylar positional changes after splint therapy in patients with myofascial pain with an average skeletal growth pattern remains limited. Therefore, the present study aimed to evaluate the effect of occlusal splint therapy combined with pharmacotherapy on mandibular condylar position in patients with M-TMDs and an average skeletal growth pattern using CBCT. Condylar ratio (CR), sagittal condylar guidance angle (SCGA), TMJ space measurements, pain intensity, and mandibular function were evaluated and compared with pharmacotherapy alone, with reference measurements obtained from healthy controls. It was hypothesized that combined occlusal splint therapy and pharmacotherapy would produce greater improvements in clinical outcomes and condylar positional parameters than pharmacotherapy alone.

## Materials and methods

Study design and setting

This two-arm, open-label randomized controlled trial, with an additional non-randomized external healthy control group, was conducted in the Department of Oral Medicine and Radiology, Bharati Vidyapeeth (Deemed to be University) Dental College and Hospital, Sangli, Maharashtra, India. Participants were recruited between April 2025 and June 2026.

Institutional Ethics Committee approval was obtained on March 1, 2025 (BVDU/IEC/R3/24-25), and the trial was prospectively registered with the Clinical Trials Registry, India on April 3, 2025 (CTRI/2025/04/084068). Following Institutional Ethics Committee permission and CTRI registration, formal participant screening and recruitment started on April 15, 2025, and lasted until August of the same year. The study was conducted in accordance with the Declaration of Helsinki and applicable institutional ethical guidelines.

Primary objective

The primary objective of this study was to evaluate treatment-associated changes in CBCT-derived mandibular condylar positional parameters, including the CR, SCGA, and TMJ space measurements, following occlusal splint therapy combined with pharmacotherapy compared with pharmacotherapy alone in patients with M-TMDs exhibiting an average skeletal growth pattern.

Secondary objectives

The secondary objectives were to assess changes in TMJ pain intensity and maximum mouth opening (MMO) following treatment and to compare these clinical outcomes between the two randomized treatment groups over the three-month follow-up period.

Sample size calculation

The sample size was estimated using OpenEpi version 3.0 data from a previous CBCT-based study evaluating changes in mandibular condylar position following occlusal splint therapy combined with physiotherapy in patients with TMD [19]. The calculation was based on a primary CBCT-derived condylar-position outcome, with standard deviations of 3.1 and 3.2 and an expected mean difference of 3.1. Considering a two-sided significance level of 5% ($Z_{1-\alpha/2}=1.96$), a statistical power of 80% ($Z_{1-\beta}=0.84$), and a 1:1 allocation ratio, the minimum required sample was calculated using the following formula:

$n=\frac{(\sigma_1^2+\sigma_2^2)(Z_{1-\alpha/2}+Z_{1-\beta})^2}{\Delta^2}$

The calculated sample size was approximately 17 patients per treatment group. After allowing for a 20% attrition rate, the required sample was increased to 22 patients per group. Accordingly, 44 patients with M-TMD were randomized equally to groups A and B. An additional 21 healthy participants aged 20-30 years were recruited separately as a non-randomized external reference group, resulting in a total sample of 65 participants.

Participant selection

Patients aged 20-30 years who were clinically diagnosed with M-TMD according to the Research Diagnostic Criteria for Temporomandibular Disorder (RDC/TMD), Axis I, were screened for eligibility. The age range of 20-30 years was selected to obtain a relatively homogeneous young-adult population and minimize potential age-related variation in TMJ morphology, degenerative changes, and craniofacial characteristics.

Inclusion/exclusion criteria

The inclusion criteria were as follows: (1) a diagnosis of myalgia or myofascial pain according to RDC/TMD Axis I, (2) an average skeletal growth pattern confirmed through composite cephalometric analysis; (3) no previous treatment for TMD, including occlusal splint therapy, occlusal rehabilitation, orthodontic treatment, or surgical treatment; and (4) willingness to provide written informed consent.

Patients were excluded if they had systemic diseases affecting the TMJ or upper airway, including rheumatoid arthritis or sleep apnea; craniofacial anomalies; degenerative TMJ condition such as osteoarthritis or ankylosis; previous orthognathic surgery; severe class II or class III skeletal malocclusion; severe open bite; respiratory disorder such as asthma, chronic obstructive pulmonary disease, or chronic rhinitis; adenoidal or tonsillar hypertrophy; deviated nasal septum; psychiatric disorder; pregnancy; or current use of medication likely to affect masticatory-muscle or TMJ function.

Healthy controls aged 20-30 years were included if they had an average skeletal growth pattern, healthy dentition, and no clinical signs or symptoms of TMD. Participants with systemic diseases, TMJ disorders, craniofacial abnormalities, respiratory conditions, previous orthognathic surgery, psychiatric disorders, pregnancy, or medication use affecting TMJ or masticatory muscle function were excluded.

Study groups

The study participants were divided into three groups. Group A (n = 22) comprised patients with M-TMD who received occlusal splint therapy combined with pharmacotherapy. Group B (n = 22) included patients with M-TMD who received pharmacotherapy alone. Group C comprised 21 non-randomized healthy participants aged 20-30 years, recruited separately from the randomized treatment groups (groups A and B). Although all three groups were selected from the same predefined age range, no formal age- or sex-matching procedure was performed. No statistical software or matching algorithms were used for control selection.

Only patients with M-TMD were randomized to groups A and B. Healthy controls were recruited separately and were not included in the random-allocation sequence.

Randomization and allocation concealment

Eligible patients with M-TMD were randomly allocated to groups A and B in a 1:1 ratio using a coin-toss method. Allocation concealment was implemented using sequentially numbered, opaque, sealed envelopes (SNOSE) prepared by the responsible investigator. The envelopes were opened sequentially only after participant enrollment and completion of the baseline assessment. Because the occlusal splint was visible, participants and treating clinicians could not be blinded to group allocation. However, the CBCT examiners and statistician were blinded to treatment allocation. Group C was not included in the process of randomization.

Clinical and cephalometric assessment

M-TMD was diagnosed using the RDC/TMD Axis I diagnostic protocol. Demographic information, clinical history, pain intensity, MMO, mandibular deviation, joint sounds, and masticatory muscle tenderness were recorded using a standard case-history proforma. 

The skeletal growth pattern was evaluated using composite cephalometric analysis performed using the Dolphin Imaging software (Dolphin Imaging & Management Solutions, Chatsworth, CA, USA). Only participants demonstrating an average skeletal growth pattern were included.

Pain intensity was recorded using an 11-point numerical rating scale ranging from 0, representing no pain, to 10, representing the worst imaginable pain. Assessments were conducted at baseline and during the scheduled follow-up visits at one, two, and three months. MMO was measured in millimeters using a graduated measuring scale. Participants were instructed to open their mouths maximally within their comfortable tolerance. The interincisal distance between the incisal edges of the maxillary and mandibular central incisors was recorded at baseline and at one-, two-, and three-month follow-up visits.

Pharmacotherapy

Participants in groups A and B received the same pharmacological regimen: one tablet of Flexon MR (ibuprofen 400 mg, paracetamol 325 mg, and chlorzoxazone 250 mg) orally twice daily for five days. Up to the recommended maximum dosage, subsequent use was only allowed as a rescue drug for breakthrough pain. A structured interview was used during follow-up to record adherence to the initial five-day treatment and any subsequent rescue use. The recommended dosage, maximum allowed intake, possible side effects, and pertinent contraindications were explained to the participants. The protocol to be followed was: Don't chew, crush, or break the tablet; instead, swallow it whole. It's crucial to take Flexon MR Tablets with food to reduce the chance of stomach upset. Do not take more than the suggested dosage. The Flexon MR Tablet frequently causes nausea, indigestion, stomach pain, weakness, lightheadedness, and tiredness. If any of these side effects were present, then the participants were offered management suggestions.

Group B just received medication, whereas group A also received a custom-fitted maxillary stabilizing splint. At the planned follow-up appointments, medication adherence was verbally evaluated by a standardized participant interview.

Occlusal splint fabrication and delivery

The appliance incorporated a flat occlusal platform designed to provide even, simultaneous bilateral occlusal contacts with the opposing mandibular teeth. Splint compliance was measured during monthly follow-up visits utilizing a structured interview approach.

Participants in group A received a custom-fabricated maxillary stabilization splint. Maxillary and mandibular impressions were obtained using alginate impression material, and dental stone casts were prepared. A 1.0-mm-thick DURAN® transparent thermoplastic sheet (SCHEU-DENTAL GmbH, Iserlohn, Germany) was adapted over the maxillary cast using a BIOSTAR® pressure-moulding machine (SCHEU-DENTAL GmbH, Iserlohn, Germany). The appliance was designed as a flat-plane maxillary stabilization splint. After thermoforming, the appliance was trimmed, finished, and polished. Intraoral adjustments were performed to obtain stability, retention, and even bilateral occlusal contact with the opposing dentition. Participants in group A were instructed to wear the appliance throughout the night while sleeping and for at least six hours during the day, for a total treatment period of three months. Splint fit, occlusal contacts, patient comfort, adverse effects, and adherence were assessed during the monthly follow-up visits. After wearing the corresponding splints for seven days, each patient was reexamined to assess their occlusal contacts and symptoms. For three months, adjustments were made every week.

The improvement or elimination of the symptoms served as the criteria for evaluating each splint's effectiveness. Three months after the splint was applied, our study evaluated symptoms.

CBCT acquisition protocol

Baseline CBCT examinations were obtained for participants in groups A-C using a CS 9600 CBCT system (Carestream Dental). Follow-up CBCT examinations were performed after three months for groups A and B. Group C underwent baseline CBCT assessment only (Figures 1-3).

**Figure 1:**
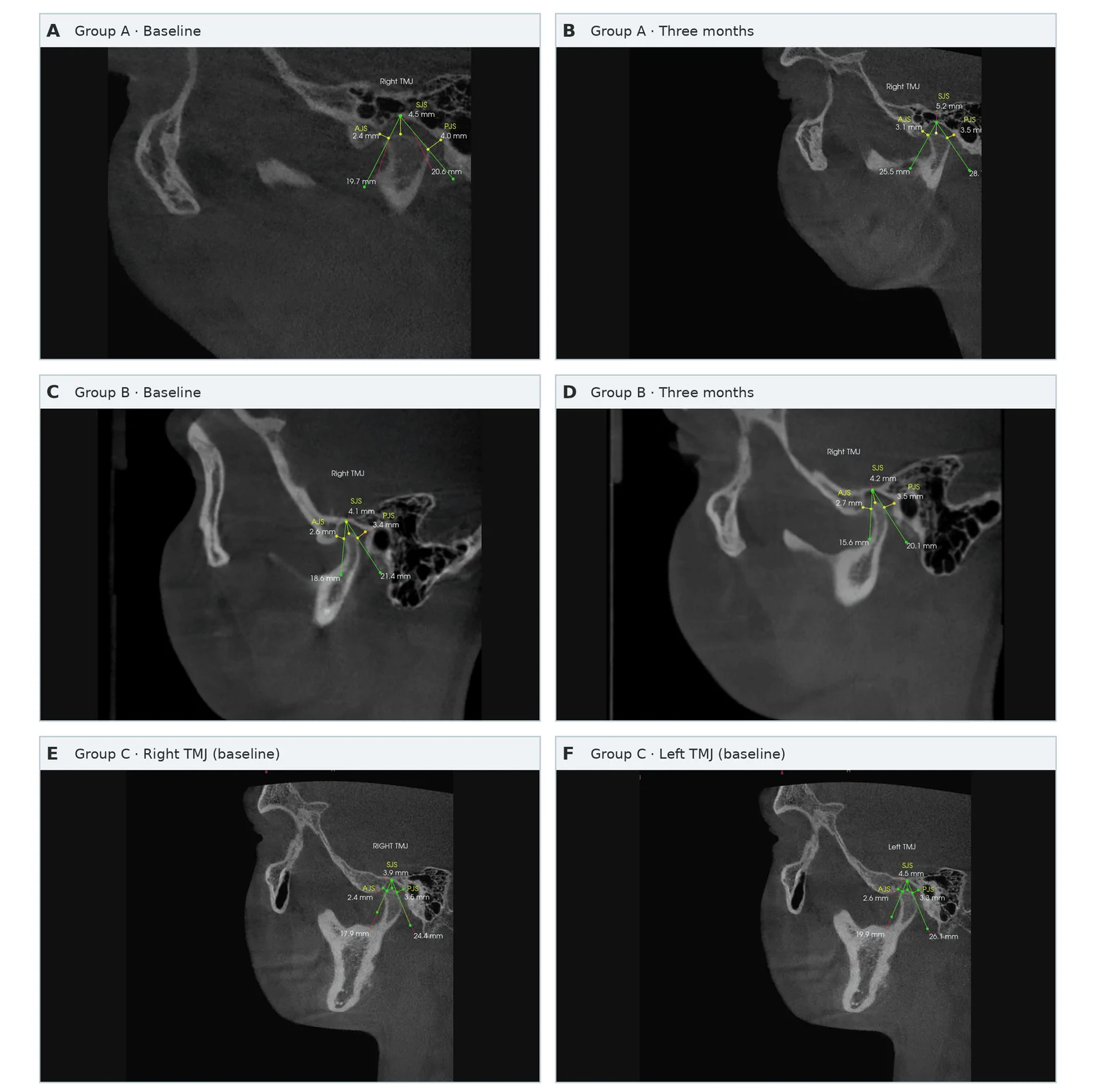
AJS, PJS, and SJS measurements of the TMJ are displayed in representative sagittal CBCT sections. (A–B) Group A (pharmacotherapy with occlusal splint therapy) at baseline and three months. (C–D) Group B (pharmacotherapy alone) at baseline and three months. (E–F) Group C (healthy reference participant's right and left TMJs) at baseline. AJS, anterior joint spaces; PJS, posterior joint spaces; SJS, superior joint spaces; CBCT, cone-beam computed tomography; TMJ, temporomandibular joint

**Figure 2:**
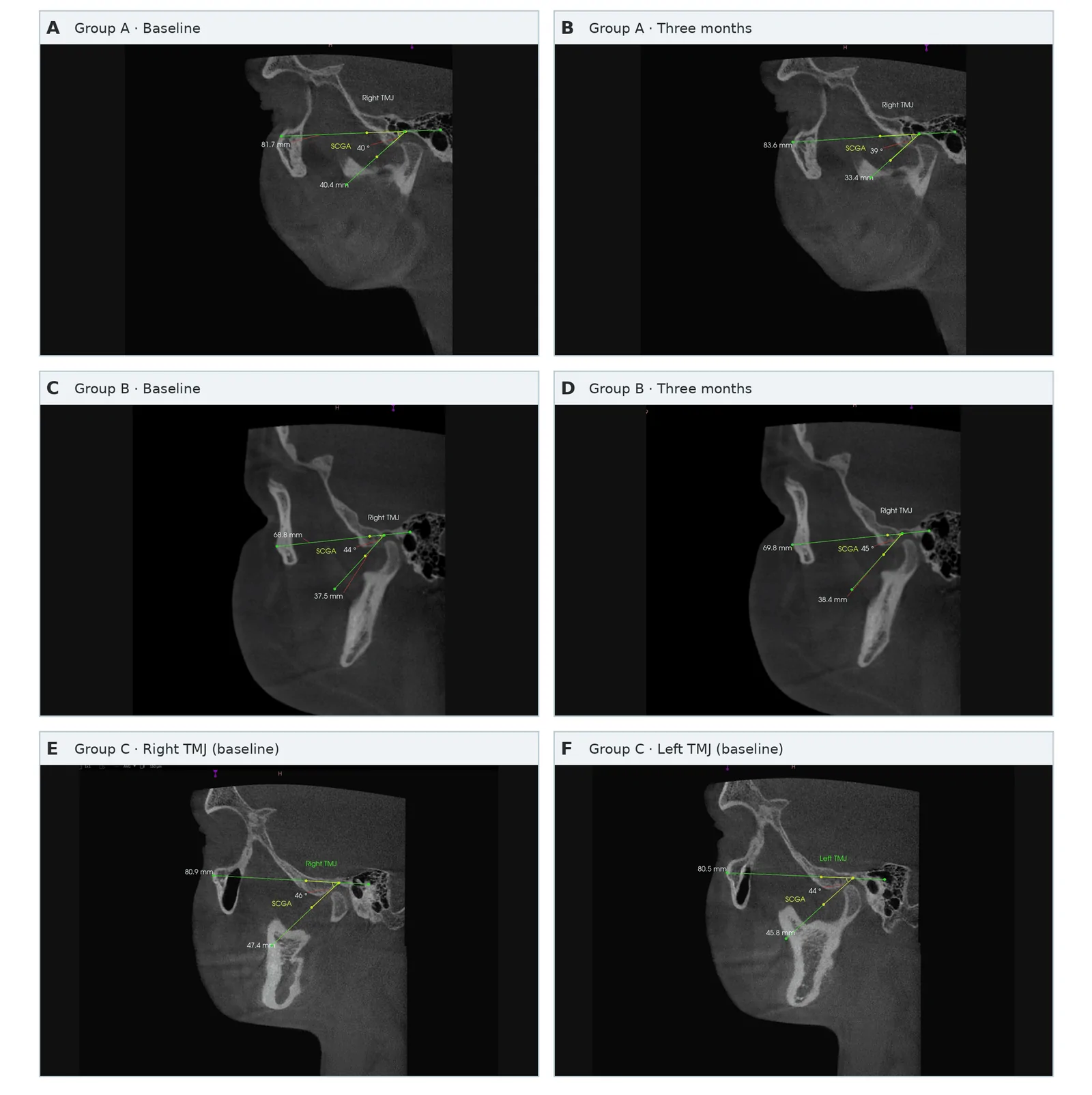
SCGA measured in representative sagittal CBCT sections. Group A at baseline and three months (40° and 39°). Group B at baseline and three months (44° and 45°). Group C (healthy reference person): right and left TMJs at baseline (46° and 44°). SCGA, sagittal condylar guidance angle; TMJ, temporomandibular joint

**Figure 3:**
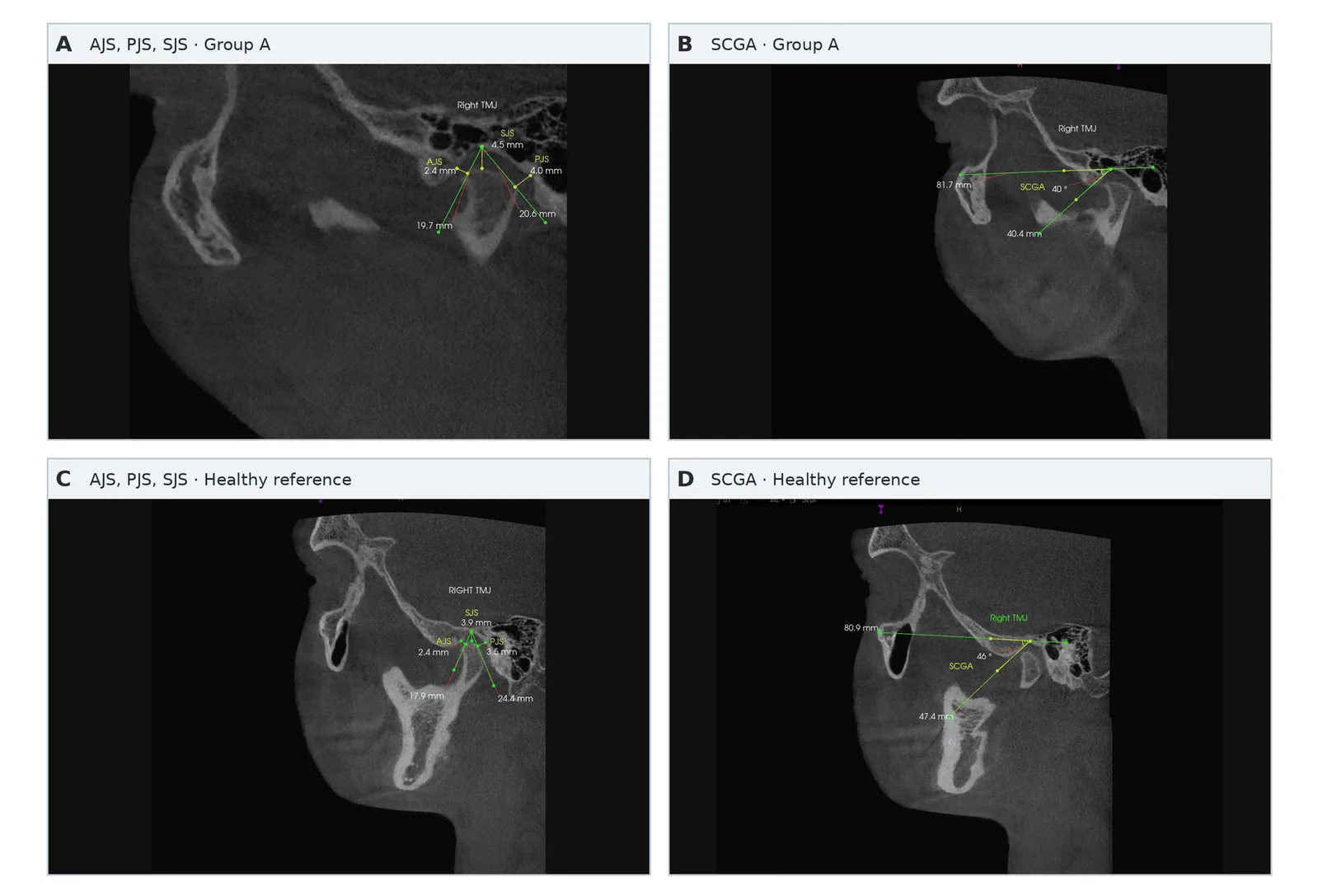
The measurement overlays utilized in the investigation are depicted in representative sagittal CBCT sections: AJS, PJS, and SJS in a group A participant (A); SCGA in a group A participant (B); AJS, PJS, and SJS in a healthy reference participant (C); and SCGA in a healthy reference participant (D). CBCT, cone-beam computed tomography; AJS, anterior joint spaces; PJS, posterior joint spaces; SJS, superior joint spaces; SCGA, sagittal condylar guidance angle

During image acquisition, each participant was positioned upright with the Frankfort horizontal plane parallel to the floor. The teeth were maintained in maximum intercuspation, the lips were relaxed, and participants were instructed to breathe through the nose and avoid swallowing during image acquisition. The exposure parameters were a 16 × 10-cm field of view, 90 kV, 10 mA, and an exposure time of 10 seconds. Images were reconstructed using CS 3D Imaging software, version 3.10.39 (Carestream Dental LLC, Atlanta, GA, USA), and exported in Digital Imaging and Communications in Medicine (DICOM) format.

CBCT assessment of condylar position

Multiplanar CBCT images were evaluated using standardized axial, coronal, and sagittal sections with a slice thickness of 0.3 mm. The axial section showing the maximum mediolateral dimension of the condylar head was identified. The medial and lateral poles of the condyle were marked, and a line connecting these landmarks was used to establish the coronal condylar axis. A perpendicular line passing through the midpoint of this axis was used to obtain the true central sagittal section. Anterior, posterior, superior, and medial joint-space measurements were obtained using standardized anatomical landmarks (Figure 1). The CR was calculated from the anterior and posterior joint space measurements using the following formula: CR = (PJS − AJS)/(PJS + AJS), where PJS represents the posterior joint space, and AJS represents the anterior joint space. The SCGA was measured bilaterally on the true central sagittal CBCT section as the angle between a line tangent to the posterior slope of the articular eminence and the Frankfort plane (Figure 2). The measurements were recorded in degrees and represented the inclination of the condylar path during mandibular movements. All CBCT measurements were obtained at baseline for the three groups and repeated after three months for groups A and B.

Reliability assessment

Before the principal analysis, five CBCT scans that were not included in the main study sample were used to assess measurement reliability. For intra-examiner reliability, the primary examiner repeated all measurements on the same scans after an interval of two weeks, while remaining blinded to the initial measurements. For inter-examiner reliability, a second trained examiner independently evaluated the same scans using an identical measurement protocol.

Both examiners underwent training and calibration before the reliability assessment. Reliability was evaluated using the intraclass correlation coefficient. The intra-examiner ICC was 0.966, and the inter-examiner ICC was 0.999, indicating excellent measurement reliability.

Statistical analysis

Statistical analysis was performed using SPSS Version 23.0 (IBM Corp., Armonk, NY). The normality of continuous variables was assessed using the Shapiro-Wilk test. Continuous variables were expressed as mean ± standard deviation (SD), whereas categorical variables were presented as frequencies and percentages.

For intergroup comparisons between the randomized treatment groups (groups A and B), the independent-samples t-test was used for normally distributed data, whereas the Mann-Whitney U test was employed for non-normally distributed data. For comparisons involving three independent groups, where applicable, one-way analysis of variance (ANOVA) followed by Tukey's post hoc test was used for normally distributed data, while the Kruskal-Wallis test with Bonferroni-adjusted post hoc comparisons was applied for non-normally distributed data.

For intragroup comparisons of baseline and post-treatment CBCT-derived condylar positional parameters, the paired-samples t-test was used for normally distributed paired data, whereas the Wilcoxon signed-rank test was employed for non-normally distributed paired data. Repeated measurements of visual analog scale (VAS) pain scores across baseline and follow-up intervals were analyzed using the Friedman test, followed by Bonferroni-adjusted post hoc pairwise comparisons.

Categorical variables, including TMJ-related symptoms, clicking sounds, mandibular deviation, muscle tenderness, and medication adherence, were summarized as frequencies and percentages. Where applicable, comparisons of categorical variables were performed using the chi-square test or Fisher's exact test. Intragroup comparisons of splint compliance within group A across follow-up periods were performed using the Friedman test, followed by Bonferroni-adjusted post hoc pairwise comparisons. A p-value of ≤0.05 was considered statistically significant.

All statistical tests were two-sided, and a p-value of ≤0.05 was considered statistically significant, with appropriate adjustments for multiple comparisons. Statistical significance was interpreted alongside the magnitude and clinical relevance of observed changes.

## Results

The mean age was 24.27 ± 1.35 years in group A, 24.14 ± 1.28 years in group B, and 24.10 ± 2.12 years in group C. Group A included 9 males and 13 females, Group B included 7 males and 15 females, and group C included 11 males and 10 females. No formal age- or sex-matching procedure was performed. Although the groups were recruited within the same age range, the sex distribution differed, with a female majority in groups A and B and a more balanced distribution in the non-randomized healthy reference group (group C).

VAS pain scores and MMO were assessed at baseline and at one-, two-, and three-month follow-up visits in the randomized treatment groups (A and B). At baseline, mean VAS scores were identical in groups A and B (7.95 ± 0.72 in each group); Group C had a VAS score of 0.00 ± 0.00 at the reported reference assessments. At one, two, and three months, mean VAS scores were 5.95 ± 0.72, 4.00 ± 0.69, and 2.27 ± 0.46 in group A, compared with 6.95 ± 0.72, 5.95 ± 0.72, and 4.91 ± 0.75 in group B, respectively. The reported intergroup comparisons were statistically significant at each follow-up (p < 0.001; Table 1).

**Table 1:** Intergroup comparison of pain (VAS score) between randomized groups (group A and group B) and non-randomized external healthy control group (group C) Data were analyzed using ^#^Mann Whitney test and ^$^Kruskal Wallis test, and Post hoc Bonferroni test. *Indicates a significant difference at p≤0.05. Randomized groups: group A (M-TMD patients + occlusal splint therapy + pharmacotherapy) and group B (M-TMD patients treated with pharmacotherapy alone). Non-randomized external healthy control group: group C. VAS, visual analog scale; SD, standard deviation; M-TMD, myogenic temporomandibular disorder

Interval	Randomized group				Non-randomized external healthy control group		Statistic	p-Value
	Group A		Group B		Group C			
	Mean	SD	Mean	SD	Mean	SD		
Baseline	7.95	0.72	7.95	0.72	0.00	0.00	45.762^$^	<0.001*
1 month	5.95	0.72	6.95	0.72	-	-	-3.768^#^	<0.001*
2 months	4.00	0.69	5.95	0.72	-	-	-5.478^#^	<0.001*
3 months	2.27	0.46	4.91	0.75	0.00	0.00	60.175^$^	<0.001*

Bonferroni-adjusted within-group pairwise comparisons showed significant differences for baseline versus two months, baseline versus three months, and one month versus three months in both randomized groups (all p < 0.001); the remaining reported pairwise comparisons were not significant (p = 0.061; Table 2).

**Table 2:** Pairwise comparison of pain (VAS score) between randomized groups (group A and group B) Data were analyzed using a post hoc Bonferroni test. *Indicates a significant difference at p≤0.05. Randomized groups: group A (M-TMD patients + occlusal splint therapy + pharmacotherapy) and group B (M-TMD patients treated with pharmacotherapy alone). VAS, visual analog scale; M-TMD, myogenic temporomandibular disorder

Pairwise comparison	Randomized group	
	Group A	Group B
Baseline vs 1 month	0.061	0.061
Baseline vs 2 months	<0.001*	<0.001*
Baseline vs 3 months	<0.001*	<0.001*
1 month vs 2 months	0.061	0.061
1 month vs 3 months	<0.001*	<0.001*
2 months vs 3 months	0.061	0.061

At baseline, mean MMO was 29.23 ± 1.60 mm in group A, 29.68 ± 1.25 mm in group B, and 42.81 ± 1.94 mm in group C (overall ANOVA p < 0.001). At one, two, and three months, mean MMO increased to 33.50 ± 1.63, 37.68 ± 1.67, and 41.50 ± 2.20 mm in group A, compared with 31.18 ± 1.65, 32.14 ± 1.62, and 33.68 ± 1.25 mm in group B. The reported comparisons between groups A and B at one and two months were significant (both p < 0.001), while the three-group comparison at three months was also significant (p < 0.001; Table 3).

**Table 3:** Intergroup comparison of maximum mouth opening (mm) at baseline and follow-up assessments among randomized treatment groups (A and B) and the non-randomized healthy reference group (C). Data were analyzed using ^#^independent t test, ^$^one-way ANOVA test, and post hoc Tukey test. *Indicates a significant difference at p≤0.05. Randomized groups: group A (M-TMD patients + occlusal splint therapy + pharmacotherapy) and group B (M-TMD patients treated with pharmacotherapy alone). Non-randomized external healthy control group: group C. SD, standard deviation; ANOVA, analysis of variance; M-TMD, myogenic temporomandibular disorder

Group	Group 1		Group 2		Group 3		Statistic	p-Value
	Mean	SD	Mean	SD	Mean	SD		
Pre	29.23	1.60	29.68	1.25	42.81	1.94	485.979^$^	<0.001*
1 month	33.50	1.63	31.18	1.65	-	-	4.692^#^	<0.001*
2 months	37.68	1.67	32.14	1.62	-	-	10.610^#^	<0.001*
3 months	41.50	2.20	33.68	1.25	42.81	1.94	146.985^$^	<0.001*

The mean MMO in group A at three months was numerically closer to the group C reference value than that in group B; however, the overall ANOVA result does not establish the significance of any specific pairwise difference. Group C was not included in the one- and two-month comparisons.

Comparison of pre- and post-treatment CR and SCGA between groups

In group A, right CR decreased from 0.24 ± 0.10 to 0.11 ± 0.04 (p < 0.001), while left CR decreased from 0.12 ± 0.23 to 0.06 ± 0.09 (p = 0.039). Right SCGA decreased from 40.18 ± 9.27° to 39.32 ± 8.17° (p = 0.005), while left SCGA decreased from 40.55 ± 8.06° to 39.09 ± 7.06° (p < 0.001). In group B, no significant pre- to post-treatment changes were observed for right CR (0.17 ± 0.19 to 0.16 ± 0.19; p = 0.285), left CR (0.18 ± 0.18 to 0.17 ± 0.18; p = 0.175), right SCGA (38.91 ± 8.65° to 39.09 ± 8.71°; p = 0.406), or left SCGA (37.86 ± 7.50° to 37.77 ± 7.34°; p = 0.680; Table 4).

**Table 4:** Intragroup comparison of CR and SCGA between randomized groups (group A and group B) Data were analyzed using a paired t-test. *Indicates a significant difference at p≤0.05. Randomized groups: group A (M-TMD patients + occlusal splint therapy + pharmacotherapy) and group B (M-TMD patients treated with pharmacotherapy alone). SD, standard deviation; CR, condylar ratio; SCGA, sagittal condylar guidance angle; M-TMD, myogenic temporomandibular disorder

Randomized group	Parameter	Pre	Post	Difference	t-Value	p-Value
		Mean±SD	Mean±SD			
Group A	CR right	0.24±0.10	0.11±0.04	0.13	9.960	<0.001*
	CR left	0.12±0.23	0.06±0.09	0.06	2.206	0.039*
	SCGA right	40.18±9.27	39.32±8.17	0.86	3.156	0.005*
	SCGA left	40.55±8.06	39.09±7.06	1.45	5.109	<0.001*
Group B	CR right	0.17±0.19	0.16±0.19	0.01	1.097	0.285
	CR left	0.18±0.18	0.17±0.18	0.01	1.405	0.175
	SCGA right	38.91±8.65	39.09±8.71	-0.18	-0.847	0.406
	SCGA left	37.86±7.50	37.77±7.34	0.09	0.418	0.680

These within-group findings indicate statistically significant changes in the measured parameters in group A but do not, by themselves, establish a statistically significant between-treatment difference or clinical importance.

Baseline comparisons among groups A, B, and the non-randomized healthy reference group (C) showed no statistically significant differences in right CR (F = 1.214, p = 0.304), left CR (F = 0.377, p = 0.687), right SCGA (F = 0.492, p = 0.614), or left SCGA (F = 2.679, p = 0.077; Table 5).

**Table 5:** Baseline intergroup comparison of CR and SCGA among the randomized treatment groups and the non-randomized healthy reference group Values are presented as mean ± SD. Intergroup comparisons were performed using ANOVA. Statistical significance was set at p ≤ 0.05. No statistically significant differences were observed among the three groups at baseline. SD, standard deviation; CR, condylar ratio; SCGA, sagittal condylar guidance angle; ANOVA, analysis of variance

Parameter	Group A (n = 22), mean ± SD	Group B (n = 22), mean ± SD	Group C (n = 21), mean ± SD	F-Value	p-Value
Right CR	0.24±0.10	0.17±0.19	0.17±0.21	1.214	0.304
Left CR	0.12±0.23	0.18±0.18	0.16±0.19	0.377	0.687
Right SCGA (°)	40.18±9.27	38.91±8.65	41.67±9.37	0.492	0.614
Left SCGA (°)	40.55±8.06	37.86±7.50	44.00±10.38	2.679	0.077

The baseline means were 0.24 ± 0.10, 0.17 ± 0.19, and 0.17 ± 0.21 for right CR; 0.12 ± 0.23, 0.18 ± 0.18, and 0.16 ± 0.19 for left CR; 40.18 ± 9.27°, 38.91 ± 8.65°, and 41.67 ± 9.37° for right SCGA; and 40.55 ± 8.06°, 37.86 ± 7.50°, and 44.00 ± 10.38° for left SCGA in groups A, B, and C, respectively. The absence of statistically significant baseline differences does not establish equivalence among groups.

At the post-treatment assessment, one-way ANOVA showed no statistically significant overall differences among groups A, B, and C for right CR (0.11 ± 0.04, 0.16 ± 0.19, and 0.17 ± 0.21, respectively; F = 1.728, p = 0.186), left CR (0.06 ± 0.09, 0.17 ± 0.18, and 0.16 ± 0.19, respectively; F = 3.083, p = 0.053), or right SCGA (39.32 ± 8.17°, 39.09 ± 8.71°, and 41.67 ± 9.37°, respectively; F = 0.566, p = 0.571). An overall difference was observed for left SCGA (39.09 ± 7.06°, 37.77 ± 7.34°, and 44.00 ± 10.38°; F = 3.291, p = 0.044; Table 6).

**Table 6:** Comparison of post-treatment CR and SCGA between randomized groups (group A and group B) and healthy control group (group C) Data were analyzed using a one-way ANOVA test. *Indicates a significant difference at p≤0.05. Randomized groups: group A (M-TMD patients + occlusal splint therapy + pharmacotherapy) and group B (M-TMD patients treated with pharmacotherapy alone). Non-randomized external healthy control group: group C. SD, standard deviation; CR, condylar ratio; SCGA, sagittal condylar guidance angle; ANOVA, analysis of variance; M-TMD, myogenic temporomandibular disorder

Parameter	Randomized group		Non-randomized external healthy control group	F-value	p-Value
	Group A	Group B	Group C		
	Mean±SD	Mean±SD	Mean±SD		
CR right	0.11±0.04	0.16±0.19	0.17±0.21	1.728	0.186
CR left	0.06±0.09	0.17±0.18	0.16±0.19	3.083	0.053
SCGA right	39.32±8.17	39.09±8.71	41.67±9.37	0.566	0.571
SCGA left	39.09±7.06	37.77±7.34	44.00±10.38	3.291	0.044*

Tukey post hoc analysis identified a significant difference in left SCGA between groups B and C (p = 0.045) but not between groups A and B (p = 0.860) or groups A and C (p = 0.140). No other pairwise comparisons were significant (Table 7).

**Table 7:** Pairwise comparison of post-treatment CR and SCGA between randomized groups (group A and group B) and non-randomized external healthy control group (group C) Data were analyzed using a post hoc Tukey test. *Indicates a significant difference at p≤0.05. Randomized groups: group A (M-TMD patients + occlusal splint therapy + pharmacotherapy) and group B (M-TMD patients treated with pharmacotherapy alone). Non-randomized external healthy control group: group C. CR, condylar ratio; SCGA, sagittal condylar guidance angle; M-TMD, myogenic temporomandibular disorder

Pairwise comparison	Group A vs group B	Group A vs group C	Group B vs group C
CR right	1.000	0.246	0.247
CR left	0.063	0.133	0.944
SCGA right	0.996	0.655	0.602
SCGA left	0.860	0.140	0.045*

Correlation between change in CR and change in total airway volume between randomized groups (group A and group B)

In group A, changes in total airway volume were negatively correlated with changes in right CR (Pearson r = -0.518, p = 0.013) and left CR (r = -0.450, p = 0.036). In group B, the corresponding correlations were not statistically significant (right CR: r = -0.027, p = 0.903; left CR: r = 0.167, p = 0.457; Table 8).

**Table 8:** Correlation between change in condylar ratio and change in total airway volume between randomized groups (group A and group B) Data were analyzed using a Pearson correlation test. *Indicates a significant correlation at p≤0.05.  Randomized groups: group A (M-TMD patients + occlusal splint therapy + pharmacotherapy) and group B (M-TMD patients treated with pharmacotherapy alone). CR, condylar ratio; M-TMD, myogenic temporomandibular disorder

Randomized group	Side	Pearson correlation coefficient	p-Value
Group A	Right CR	-0.518	0.013*
	Left CR	-0.450	0.036*
Group B	Right CR	-0.027	0.903
	Left CR	0.167	0.457

Correlation between change in condylar ratio and change in oropharyngeal volume between randomized groups (group A and group B)

Spearman rank correlation analysis showed no statistically significant associations between changes in oropharyngeal volume and changes in CR in either treatment group. In group A, the correlations were ρ = -0.241 (p = 0.280) for right CR and ρ = -0.281 (p = 0.205) for left CR. In group B, they were ρ = 0.102 (p = 0.651) for right CR and ρ = 0.037 (p = 0.872) for left CR (Table 9).

**Table 9:** Correlation between change in condylar ratio and change in oropharyngeal volume between randomized groups (group A and group B) Data were analyzed using Spearman rank correlation test. Randomized groups: group A (M-TMD patients + occlusal splint therapy + pharmacotherapy) and group B (M-TMD patients treated with pharmacotherapy alone). CR, condylar ratio; M-TMD, myogenic temporomandibular disorder

Randomized group	Side	Spearman’s rho	p-Value
Group A	Right CR	-0.241	0.280
	Left CR	-0.281	0.205
Group B	Right CR	0.102	0.651
	Left CR	0.037	0.872

Correlation between change in CR and change in minimum cross-sectional area (CSA) in groups A and B: neither group showed a statistically significant Pearson correlation between change in CR and change in minimum CSA. In group A, the correlations were r = -0.384 (p = 0.077) for right CR and r = -0.418 (p = 0.053) for left CR. In group B, the corresponding correlations were r = 0.243 (p = 0.275) and r = 0.007 (p = 0.974; Table 10).

**Table 10:** Correlation between change in condylar ratio and change in minimum CSA between randomized groups (group A and group B) Data were analyzed using a Pearson correlation test. Randomized groups: group A (M-TMD patients + occlusal splint therapy + pharmacotherapy) and group B (M-TMD patients treated with pharmacotherapy alone). CR, condylar ratio; CSA, cross-sectional area; M-TMD, myogenic temporomandibular disorder

Randomized group	Side	Pearson correlation coefficient	p-Value
Group A	Right CR	-0.384	0.077
	Left CR	-0.418	0.053
Group B	Right CR	0.243	0.275
	Left CR	0.007	0.974

## Discussion

The present study evaluated the efficacy of occlusal splint therapy, individually or in combination with pharmacotherapy, on mandibular condyle position in patients with M-TMDs and average skeletal growth patterns in the management of M-TMDs. Given the interdependence of craniofacial function, mandibular dynamics, and respiratory physiology, it is especially important to comprehend the link between occlusal splint therapy and airway dimensions. Furthermore, differences in the vertical and anteroposterior craniofacial skeletal patterns are known to affect TMJ biomechanics, mandibular condyles, and upper airway size. As a result, the present study limited samples to those with typical skeletal growth patterns, lowering confounding variability and improving the study's internal validity.

In the present study, M-TMDs were included since this type of TMD is the most common type encountered in the general population, affecting about 85% with an overall prevalence of 45% [20]. The study reported that this type results in localized myalgia, referred pain, motor dysfunction or autonomic responses, and discomfort with 74% of trigger points (latent or active), resulting in a tightened band of skeletal muscle. Hence, these individuals displayed a depressive state-like condition with reduced activities of daily living (ADLs) and an overall lower health-related quality of life (HRQoL) [21].

The intensity of pain decreased, and mouth opening improved from the initial visit to the three-month follow-up with combination therapy in the current study. Some comparative studies indicated that application of combination therapy was much more effective than monotherapy alone and demonstrated how myofascial pain associated with TMDs is multifaceted, involving behavioral, joint, and muscle components [22-25]. This could be attributed to the fact that an occlusal splint acts as a pacifier and reduces the occlusal load on both the muscular component and the TMJ, thereby reducing pain and increasing mouth opening in these cases. By acting as a mechanical buffer, occlusal splints provide a flat, even, customized surface, ensuring bilateral simultaneous contact and equal distribution of masticatory forces across the upper and lower jaw. Along with other therapies, it successfully addresses rehabilitation of the entire stomatognathic system, resulting in greater pain reduction and restoration of jaw motion.

In the present study, a 1.0-mm DURAN® transparent thermoplastic sheet was used as it provided consistent, synchronous contacts with the opposing dentition in a centric relationship, resulting in muscle relaxation and occlusal stability when compared to other types of stabilization splints. The results were in accordance with other contemporary studies [26,27].

In the present study, occlusal splint therapy significantly improved the mandibular condylar dimensions. The results were in alignment with other studies [28-30]. Since the mandibular condyle plays an important role in maintaining the harmony between TMJ spaces, eccentric condyle position in the glenoid fossa, and articular disc placement, the importance of analysis of dimensional changes at both condylar poles can provide further insights into positional changes that can occur during splint therapy. The observed diversity in condylar response, however, emphasizes the necessity for individualized treatment plans and additional study to comprehend the processes and long-term implications of splint-induced positional changes.

In the present study, one tablet of Flexon MR (ibuprofen 400 mg, paracetamol 325 mg, and chlorzoxazone 250 mg) orally twice daily for five days was prescribed for group B. Significant improvements were noted in group B participants since this medication neutralizes the aggressive inflammatory environment, resulting in condyle transitions from a degenerative-active state to a degenerative-repair state.

The present study reported a significant correlation between CR and SCGA, indicating positional changes in condylar dimensions. The relationship of the mandibular condyle is mainly mechanical and anatomical. The healthy reference group was non-randomized and differed numerically in sex distribution from the treatment groups. Baseline comparisons with group C were not presented in the submitted analysis. Small angular changes, such as the 0.86° right SCGA reduction, may fall within measurement variability; an absolute measurement-error estimate was not reported. The trial does not establish anatomical normalization or long-term remodeling. The condyle controls the position and rotation of the lower jaw. The study indicated that the condylar concentricity and position can aid in determining the direct mechanical relationship.

A strong association between CR and SCGA, which indicates positional changes in condylar dimensions, was found in this study. The mandibular condyle primarily has an anatomical and mechanical link. The lower jaw's rotation and position are managed by the condyle. According to the study, detecting the direct mechanical relationship can be aided by condylar concentricity and location [30].

The present study has several limitations. The relatively small sample size may restrict power and generalizability. The three-month follow-up cannot establish long-term positional stability. The open-label design may introduce performance or reporting bias despite blinded CBCT assessment and statistical analysis. Group C was non-randomized, and demographic differences may confound comparisons. The unequal sex distribution between the randomized treatment groups and the non-randomized healthy reference group represents a potential limitation, as sex-related differences may influence TMJ morphology and condylar positional measurements. Therefore, comparisons involving the healthy reference group should be interpreted cautiously. Finally, CBCT provides static osseous imaging and cannot directly evaluate disc position, dynamic function, or soft-tissue adaptations. Changes should therefore be interpreted as short-term positional adaptations rather than definitive structural remodeling or normalization. Although clinical parameters such as mandibular deviation, deflection, joint sounds (clicking/crepitus), muscle tenderness, and TMJ-related complaints are important indicators of TMD-related dysfunction, they were not designated as primary outcome measures in the present study. In order to make it easier to draw conclusions about the effectiveness of therapy, patients with primarily basic signs and symptoms of just M-TMD were chosen. The QoL assessment standards were not objectively assessed, and the possible adverse effects of occlusal splint therapy could have been systematically documented as a feedback follow-up mechanism. All three groups had different follow-up plans and protocols. Other limitations included a small single-center sample, short follow-up, open-label design, lack of a sham splint, non-randomized healthy controls, different imaging schedules, concomitant medication, multiple comparisons, no MRI/EMG, static upright airway imaging, and limited generalizability.

Clinical implications

Occlusal splint therapy consistently reduces masticatory muscle discomfort and spontaneous pain intensity as observed via VAS scores. It additionally aids in neuromuscular activity modification by lowering electromyographic activity in the masseter and anterior temporalis muscles and promoting muscle relaxation and improvement in mouth opening. Furthermore, it helps in occlusal harmonization by evenly distributing occlusal stresses throughout the dental arch and relieves strain on retrodiscal tissues by creating a centric relation occlusion and removing posterior interferences.

## Conclusions

In patients with M-TMDs and an average skeletal growth pattern, three months of occlusal splint therapy combined with pharmacotherapy was associated with reduced pain intensity, increased MMO, and significant within-group changes in CBCT-derived condylar positional parameters. However, most post-treatment intergroup comparisons of CR and SCGA were not statistically significant. The observed CBCT findings should therefore be interpreted as short-term positional adaptations rather than definitive evidence of condylar remodeling. Larger, multicenter randomized controlled trials with blinded outcome assessment and longer follow-up are required to confirm the clinical significance and long-term stability of these findings.
